# Perindopril and a Galectin-3 Inhibitor Improve Ischemic Heart Failure in Rabbits by Reducing Gal-3 Expression and Myocardial Fibrosis

**DOI:** 10.3389/fphys.2019.00267

**Published:** 2019-03-22

**Authors:** Sha Li, Shuren Li, Xiao Hao, Yuehua Zhang, Wenhao Deng

**Affiliations:** ^1^Department of Examination Center, Hebei General Hospital, Shijiazhuang, China; ^2^Department of Cardiovascular Division 1, Hebei General Hospital, Shijiazhuang, China

**Keywords:** galectin-3 inhibitor, heart failure, myocardial infarction, ventricular remodeling, perindopril

## Abstract

**Objective:** Ventricular remodeling is considered the basis of heart failure and is involved in myocardial fibrosis. This study aimed to assess perindopril and a galectin-3 inhibitor (modified citrus pectin, MCP) for their effects on ventricular remodeling and myocardial fibrosis in rabbits with ischemic heart failure.

**Methods:** Rabbits were divided into sham, heart failure (model), MCP, and perindopril groups, respectively. A rabbit model of ischemic heart failure was established by ligating the anterior descending coronary artery. Then, the rabbits were orally administered MCP, perindopril, or saline (all at 2 ml/kg/d) for 4 weeks. Sham animals only underwent open heart surgery without further treatment. After 4 weeks, cardiac function was examined by ultrasound, and myocardial Gal-3, collagen type I, and collagen type III expression was assessed, at the gene and protein levels, by real-time PCR and Western-Blot, respectively; serum Gal-3 was detected by ELISA, and fibrosis in the infarct zone was evaluated by H&E and Masson staining.

**Results:** In model animals, myocardial Gal-3, collagen type I, and collagen type III gene and protein expression levels were increased compared with control values, as well as serum Gal-3 amounts. Treatment with perindopril and MCP significantly alleviated the above effects, with no significant differences between the treatment groups. Pathological analyses showed that compared with model animals, treatment with MCP or perindopril resulted in relatively neatly arranged myocardial cells in the infarct zone, with significantly decreased fibrosis.

**Conclusion:** Perindopril and the galectin-3 inhibitor MCP comparably improve ischemic heart failure in rabbits, by downregulating Gal-3 and reducing myocardial fibrosis.

## Introduction

After Myocardial Infarction (MI), a large number of myocardial cell necrosis, hyperplasia of fibrous tissue, scar formation, as the consequent process to cardiac the adaptive dynamic the processes to the as cardiac apoptosis, angiogenesis, fibrosis, and hypertrophy and ventricular remodeling and cardiac function decline, often appear in late refractory heart failure (Marfella et al., 2013a; Sardu et al., 2014, 2016). The cardiac remodeling during HF is the resulting final process of multiple epigenetics, molecular and cellular processes involving different phases of cardiac adaptive response to pathogenic alterations of pre-load and post-load, and then leading to substantial alterations in mechanical cardiac muscle properties (diastolic and systolic altered cardiac properties) also in conditions of non-ischemic HF (Vicario et al., 2006). Ventricular remodeling is considered the basis for the development of heart failure (Kalyanasundaram et al., 2013). Meanwhile, myocardial fibrosis plays an important role in the incidence and development of ventricular remodeling and heart failure (Chen et al., 2012; Psarras et al., 2012). In recent years, delaying the progression of ventricular remodeling has become the main direction for heart failure treatment (Yancy et al., 2017). Currently, the associations of Galectin-3 (Gal-3) with heart failure and ventricular remodeling attract increasing attention (Amin et al., 2017; Baggen et al., 2017; van Vark et al., 2017). Gal-3, a soluble β-galactoside-binding protein, is widely distributed in the heart, kidney, liver, lung, and gut, and highly expressed in activated macrophages, basophils, and mast cells (De Boer et al., 2010). A recent study demonstrated that Gal-3 promotes myocardial fibrosis, and participates in the processes of myocarditis and ventricular remodeling (Meijers et al., 2015). Therefore, Gal-3 might constitute a new indicator of heart failure, and could be used for its diagnosis and prognosis. Based on the above, inhibiting Gal-3 could become a new direction in the treatment of heart failure.

As the cornerstone of clinical treatment of heart failure, angiotensin-converting enzyme inhibitors (ACEIs) improve ventricular remodeling (Wei et al., 2016). However, whether ACEIs improve ventricular remodeling in rabbits with ischemic heart failure in association with Gal-3 downregulation remains unclear. Currently, no myocardial fibrosis inhibitor is used directly to treat clinical heart failure. Therefore, this study aimed to assess the effects of modified citrus pectin (MCP, Gal-3 inhibitor) and the long-acting ACEI perindopril on ventricular remodeling and myocardial fibrosis in rabbits with ischemic heart failure. After heart failure modeling, treatment with MCP or perindopril resulted in alleviated myocardial infarction by improving myocardial fibrosis.

## Materials and Methods

### Animal Model Establishment and Treatment

A total of 40 male healthy 3-month-old New Zealand rabbits (Certification No.: 1606173; 2.5 ± 0.2 kg) were provided by the experimental animal center of Hebei Medical University. The animals were housed in specific-pathogen-free (SPF) conditions. After 10 days of adaptation, the rabbits were randomly divided into sham operation, heart failure (model), MCP, and perindopril groups (*n* = 10/group). The animals were anesthetized by 3% pentobarbital (1 ml/kg) injection in the auricular vein with a venous indwelling needle. After disappearance of corneal reflex and muscle tension, the rabbits were fixed in the supine position, followed by chest skin shaving and disinfection. The rabbit model of ischemic heart failure was established by open heart surgery and ligating the anterior descending coronary artery (Xiao et al., 2016). The sham operation group underwent open heart surgery only. All rabbits received intramuscular injection of penicillin daily for consecutive 3 days. In postoperative electrocardiography, the anterior descending coronary artery was ligated. The apex of the heart as well as the anterior wall of the left ventricle changed from red to dark purple; meanwhile, local myocardial motion was decreased, with precordial ST-segment elevation and left ventricular ejection fraction (LVEF) reduced by more than 50% 2 weeks after operation, indicating the model was successfully established (De Boer et al., 2010). Afterward, the MCP and perindopril groups were gavaged with MCP at 0.15 mg/kg/d and perindopril at 0.66 mg/kg/d, respectively, in normal saline (2 ml/kg/d); the sham operation and model groups received normal saline only (2 ml/kg/d). The treatments lasted for 4 weeks.

### Echocardiography

Left ventricular ejection fraction and left ventricular end diastolic diameter (LVEDD) were measured by echocardiography (TOSHIBA, Tokyo, Japan).

### Real-Time PCR

After treatment, myocardial tissue specimens from the infarct zone were harvested and homogenized. The TRIzol one step method (Thermo Fisher Scientific, Waltham, MA, United States) was used for total RNA extraction; agarose gel electrophoresis was used to determine RNA integrity. Reverse transcription was carried out with SuperScript III First-Strand cDNA System for RT-PCR (Thermo Fisher Scientific) following the manufacturer’s instructions. Then, real-time fluorescent quantitative PCR detection kit (Thermo Fisher Scientific) was used to assess the gene expression levels of collagen type I, collagen type III, and Gal-3. PCR was performed at 96°C for 4 min, followed by 45 cycles of 95°C for 15 s and 60°C for 60 s, and a final elongation at 60°C for 5 min. The following primers were used: actin, forward 5′-AGATCGTGC GGGACATCAAG-3′ and reverse 5′-CAGGAAGGAGGGCTG GAAGA-3′; collagen type I forward 5′-TGAGCCAGCAGATTGAGAACAT-3′ and reverse 5′-TGTCGCAGAAG ACCTTGATGG-3′; collagen type III forward 5′-GTACAACTAGCATTCCTCCGACTG-3′ and reverse 5′-TTAGAGCAGCCATC CTCCAGAAC-3′; Gal-3 forward 5′-CTGTGCCTTATGACCTGCCTCT-3′ and reverse 5′-TCATTGACCGCA ACCTTGAAGTG-3′. Data analysis was performed by the 2^-ΔΔCt^ method, with actin as a reference gene.

### Western Blot

After treatment, tissue samples from the marginal zone of myocardial infarction (50 mg) were homogenized, and lysed with RIPA lysis buffer (Beyotime, China). Protein concentration was analyzed with Pierce^TM^ BCA Protein Assay Kit (Thermo Fisher Scientific). Equal amounts of protein were separated by 8–12% SDS-PAGE and transferred onto PVDF membranes (Bio-Rad, Hercules, CA, United States). After blocking of non-specific signals by 5% (v/v) skim-milk, the membranes were incubated with primary antibodies targeting actin, collagen type I, collagen type III, and Gal-3, respectively, at 4°C overnight. Next, donkey anti-goat-HRP and sheep anti-mouse-HRP (1:5000) secondary antibodies were added for 50 min at room temperature. Detection was performed with Pierce^TM^ ECL Western Blotting Substrate (Thermo Fisher Scientific). The QuantityoneV4.52 software (Bio-Rad) was used for the quantitation of immunoreactive bands. Relative protein expression levels were determined based on actin expression in the same sample.

### Enzyme-Linked Immunosorbent Assay (ELISA)

Arterial blood was collected from the middle ear of the rabbits, and centrifuged for 15 min, for serum collection. Serum Gal-3 levels were assessed with a specific ELISA kit (Shanghai BlueGene Biotech Co., Ltd., Shanghai, China), according to the manufacturer’s instructions.

### Histopathology

After treatment, the animals were sacrificed, and the hearts were harvested and rinsed with ice cold saline. Myocardial tissues were isolated, fixed in paraformaldehyde and paraffin embedded. The sections were sliced at 5 μm and submitted to hematoxylin-eosin (H&E) and Masson staining. The stained slices were observed under a light microscope (Tokyo, Japan).

### Statistical Analysis

The SPSS 21.0 software (SPSS, Chicago, IL, United States) was used for statistical analyses. Normally distributed measurement data were presented as mean ± standard deviation (SD). Non-normally distributed data were expressed in median (quartile). Single factor analysis of variance (*one-way ANOVA*) for completely random design was used to compare normally distributed data, with the Student–Newman–Keuls (SNK)*-q* test employed for multiple comparisons. The *Kruskal–Wallis-H* test was used for non-normally distributed data. Two-sided *P* < 0.05 was considered statistically significant.

## Results

### MCP and Perindopril Improve Heart Failure in the Rabbit Model

A total of 33 rabbits survived after successful modeling. In the sham operation group, 8 rabbits survived; one animal died of massive bleeding perioperatively and another of pneumothorax during chest opening. In the model group, 8 animals survived and 2 died of ventricular fibrillation. In the MCP group, 9 animals survived and 1 died of pneumothorax; 8 rabbits survived in the perindopril group, while one each died of pneumothorax during chest opening and ventricular fibrillation induced by the ligation of the anterior descending coronary artery.

There were no statistically significant differences in LVEF (Table 1) and LVEDD (Table 2) among groups before operation (*P* > 0.05). Two weeks after the surgical operation, LVEF and LVEDD significantly differed among groups (all *P* < 0.05), with increased LVEF and reduced LVEDD in animals with heart failure (control, MCP, and perindopril groups) compared with the sham group. At 4 weeks of treatment, the MCP and perindopril groups showed markedly increased LVEF and decreased LVEDD in comparison with heart failure (*P* < 0.05) (Tables 1, 2). Meanwhile, there were no statistically significant differences in LVEF and LVEDD between the MCP and perindopril groups (*P* > 0.05).

**Table 1:** Left ventricular ejection fraction pre-operation, 2 weeks post-operation, and 4 weeks after drug administration (mean ± SD%).

Group	Sham operation	Heart failure (model)	MCP (Gal-3 inhibitor)	Perindopril
Before operation	69.12 ± 1.77	70.88 ± 2.19	71.61 ± 1.24	72.78 ± 6.21
2 weeks after operation	67.83 ± 1.94	49.17 ± 1.27^a^	49.09 ± 0.89^a^	48.60 ± 0.99^a^
4 weeks after medication	61.37 ± 5.45	40.22 ± 0.87^a^	59.96 ± 3.14^b^	57.81 ± 3.89^b^


*Left ventricular ejection fraction (LVEF); ^*a*^, *P* < 0.05 vs. Sham operation group; ^*b*^, *P* < 0.05 vs. heart failure group*.

**Table 2:** Left ventricular end diastolic diameter pre-operation, 2 weeks after operation, and 4 weeks after drug administration (mean ± SD mm).

Group	Sham operation	Heart failure (model)	MCP (Gal-3 inhibitor)	Perindopril
Before operation	10.08 ± 0.41	10.38 ± 0.31	10.83 ± 0.71	10.84 ± 0.83
2 weeks after operation	11.30 ± 0.25	14.14 ± 0.49^a^	14.75 ± 0.67^a^	14.89 ± 0.49^a^
4 weeks after medication	11.82 ± 0.31	16.59 ± 0.55^a^	12.57 ± 1.45^b^	12.66 ± 0.67^b^


*Left ventricular end diastolic diameter (LVEDD); ^*a*^, *P* < 0.05 vs. Sham operation group; ^*b*^, *P* < 0.05 vs. heart failure group*.

### MCP and Perindopril Significantly Downregulate Gal-3, Collagen Type I, and Collagen Type III

**FIGURE 1:**
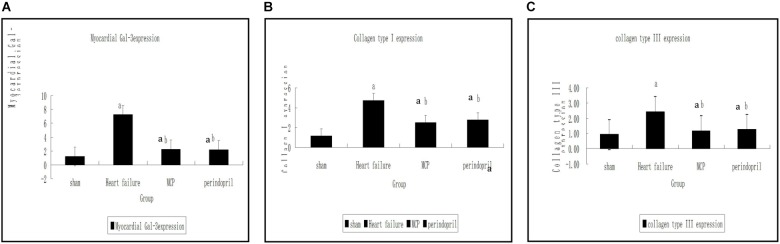
Gene expression levels of Gal-3, collagen type I, and collagen type III in myocardial tissue samples from the infarction zone. **(A–C)** Gene expression was assessed by RT-PCR in sham, model, MCP, and perindopril groups, at the indicated times. a, *P* < 0.05 vs. Sham operation group; b, *P* < 0.05 vs. heart failure group.

Compared with the sham operation group, significantly increased mRNA expression levels of collagen type I, collagen type III, and Gal-3 were found in the model, MCP, and perindopril groups (*P* < 0.05, Figure 1). Compared with the model group, the MCP and perindopril groups showed significantly decreased mRNA expression levels of collagen type I, collagen type III, and Gal-3 (*P* < 0.05, Figure 1). However, no statistically significant differences were found between the MCP and perindopril groups (*P* > 0.05, Figure 1).

**FIGURE 2:**
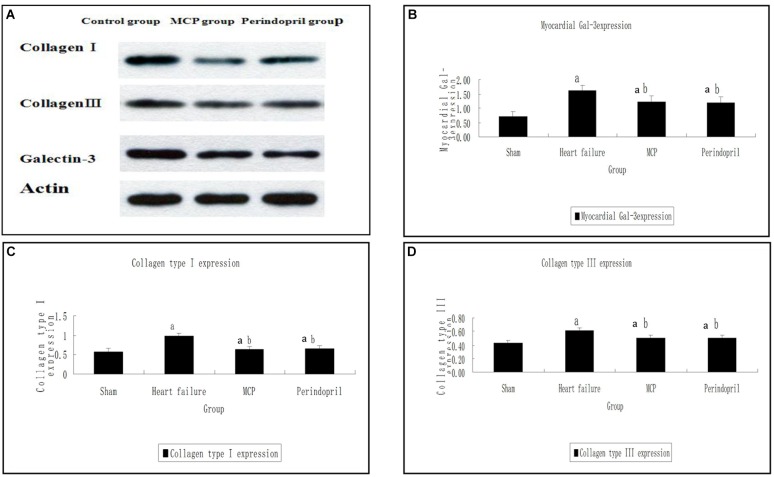
Protein contents of Gal-3, collagen type I, and collagen type III in myocardial tissue samples from the infarction zone. **(A)** Western-Blot results of collagen type I, collagen type III, and Gal-3; **(B–D)** Protein expression levels were assessed by immunoblot in sham, model, MCP, and perindopril groups, at the indicated times. ^a^, *P* < 0.05 vs. Sham operation group; ^b^, *P* < 0.05 vs. heart failure group.

Similarly, Western-Blot showed that collagen type I, collagen type III, and Gal-3 protein amounts in the model, MCP, and perindopril groups were increased compared with the values obtained for sham animals (*P <* 0.05, Figure 2). In comparison with the model group, the myocardial protein contents of collagen type I, collagen type III, and Gal-3 were decreased significantly after treatment with MCP and perindopril, respectively (*P <* 0.05, Figure 2). However, no statistically significant differences were found between the MCP and perindopril groups (*P* > 0.05, Figure 2). Gal-3 levels were comparable among the three groups preoperatively (*P* > 0.05, Figure 2).

### Changes in Circulatory Gal-3 Levels

**FIGURE 3:**
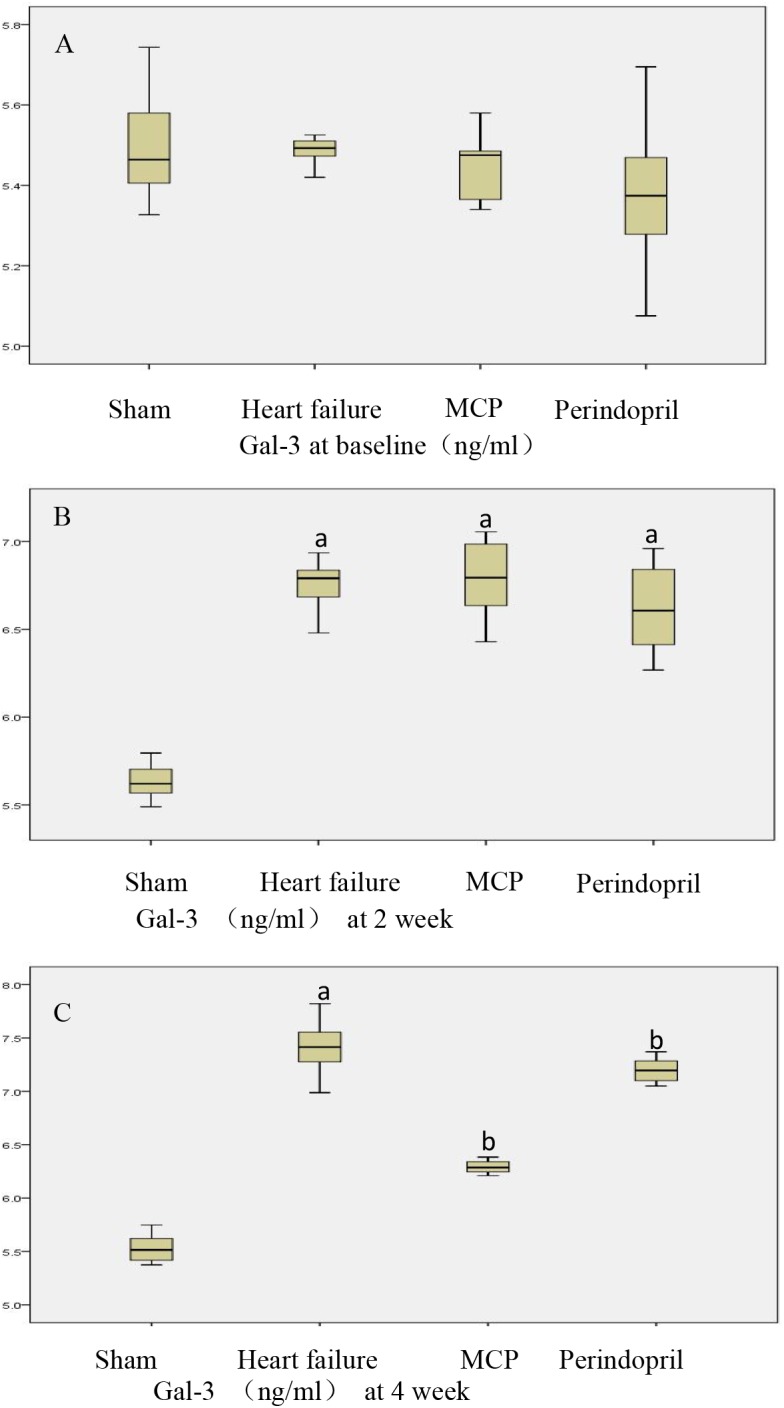
Serum Gal-3 levels pre-operation, 2 weeks post-operation, and 4 weeks after drug administration. **(A–C)** Serum Gal-3 levels were assessed by ELISA in sham, model, MCP, and perindopril groups, at the indicated times. ^a^, *P* < 0.05 vs. Sham operation group; ^b^, *P* < 0.05 vs. heart failure group.

Next, serum levels of Gal-3 were assessed. Compared with the sham operation group, the model, MCP, and perindopril groups showed significantly increased serum Gal-3 levels 2 weeks postoperatively (*P* < 0.05). These levels remained significantly higher in the model group compared with the sham operation group at 4 weeks (*P* < 0.05). Meanwhile, Gal-3 levels were markedly reduced after treatment with MCP and perindopril, in comparison with the model group (*P* < 0.05). However, the MCP and perindopril groups showed similar Gal-3 levels (Figure 3).

### Histopathological Findings

**FIGURE 4:**
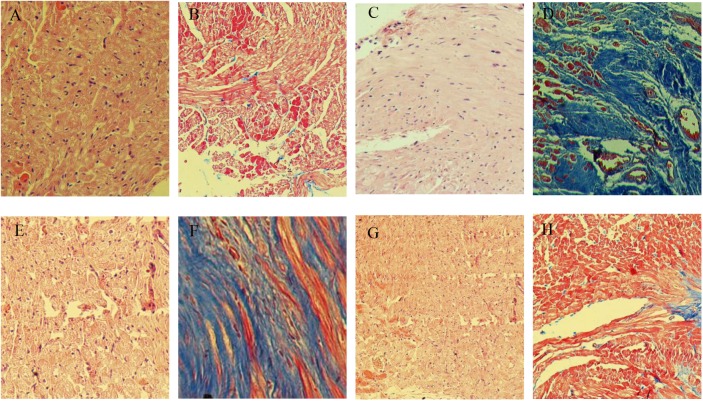
Representative H&E and Masson staining micrographs. **(A,C,E,G)**, H&E staining of specimens from sham, model, MCP, and perindopril groups, respectively. **(B,D,F,H)**, Masson staining of specimens from sham, heart failure, MCP, and perindopril groups, respectively (magnification × 100).

In sham operated rabbits, the myocardial tissues showed homogeneous and orderly arranged cells; the cytoplasm was uniformly stained, with nuclei similar in size and oval or round in shape, in the center of the cytoplasm (Figure 4A). In the model group, myocardial tissues showed cells of different in sizes and disorderly arranged; cytoplasmic staining was uneven and scattered, with no or shuttled nuclei (Figure 4C). Meanwhile, cardiomyocyte morphologies in the MCP and perindopril groups were relatively more regular and neatly arranged, with rarely scattered nuclei (Figure 4E,G).

Masson staining showed that myocardial cells in the sham operation group were homogeneous, and stained red evenly, with limited blue fibrous tissues observed (Figure 4B). In the model group, myocardial cells in the infarct zone disappeared, and were replaced by fibroblasts and large amounts of fibrous collagen bundles, which were loosely arranged (Figure 4D). Compared with the perindopril group, fibrous hyperplasia in the MCP group was reduced, with myocardial cells neatly arranged (Figure 4H,F).

## Discussion

Modified citrus pectin such as perindopril treatments are both involved in the regulation of cardiac fibrosis as synthesis, relapse, extension in myocardial cells and secondary as alterations of myocardial structure. An initial clinical observations indicated Gal-3 as biomarker for decompensated heart failure, with incremental value over well-used “pressure-dependent” biomarkers, such as B-type natriuretic peptide (De Boer et al., 2010). Gal-3 plays an important role in many cardiovascular diseases, participating in myocardial fibrosis, and promoting ventricular remodeling and heart failure (McCullough et al., 2011; de Boer et al., 2012; Lok et al., 2013). meanwhile, Gal-3 might play a relevant role in HF pathogenic characterization and diagnosis, such as in the prognostic evaluation of worse clinical events (deaths, hospitalization for decompensed HF events, arrhythmias etc.), parallel to old and well known HF biomarkers as BNP, and new HF biomarkers as ST2 protein (Petretta et al., 2007; Katsanos et al., 2017; Sardu et al., 2018b). In a hyperaldosteronism test, Calvier et al. (2015) found that increased expression of Gal-3 is associated with cardiac and renal fibrosis, which is alleviated by drug inhibition, inhibition of Gal effects, or Gal-3 gene alterations. Vergaro et al. (2016) found that the Gal-3 inhibitor MCP and aldosterone inhibitor reverse isoprenaline associated ventricle dysfunction, and improve ventricular remodeling by reducing the occurrence of myocarditis and fibrosis. Whether ACEI, an upstream inhibitor of aldosterone, could further alleviate myocardial fibrosis and improve ventricular remodeling by inhibiting Gal-3 is unclear, as well as how ACEIs compares with Gal-3 inhibitors in inhibiting myocardial fibrosis. Intriguingly, Gal-3 protein properties might match between the clinical application of myocardial stretching biomarkers (as BNP and ST2) and myocardial damage biomarkers (as troponin), and this might more link the cardiac fibrosis to a broad spectrum of cardiac adaptive processes from cardiac inflammation toward cardiac stretching and apoptosis (Katsanos et al., 2017; Sardu et al., 2018b). Currently, no clinical study has assessed the direct improvement of myocardial fibrosis in ischemic heart failure by the Gal-3 inhibitor MCP. In this study, ischemic heart failure was improved by MCP.

By pathological examination, we demonstrated that treatment with MCP and perindopril resulted in relatively regular and neatly arranged cardiac myocytes, with nuclei rarely scattered, as well as markedly decreased fibrous hyperplasia. Meanwhile, real-time PCR and Western-blot also showed that after treatment with MCP and perindopril, the mRNA and protein levels of Gal-3 in myocardial tissue specimens from the infarct zone were significantly decreased, as well as collagen types I and III amounts. The effects of MCP and perindopril were comparable, indicating that perindopril improves ventricular remodeling in rabbits with ischemic heart failure probably by downregulating Gal-3 in the myocardium, decreasing collagen deposition in the myocardial interstitium, inhibiting myocardial fibrosis, and delaying ventricular remodeling. These findings provide an experimental basis for the direct application of Gal-3 inhibitors in clinical practice.

In long term studies on cardiac structure and function changes in individuals with heart failure, Baumbach and Heistad (1989) first proposed in 1989 the concept of ventricular remodeling, which refers to heart damage under the comprehensive effects of hemodynamic and neurohumoral factors, with abnormal gene expression leading to changes in structure, metabolism and function, including structural, functional, and electrical reconfigurations. The manifestations of ventricular remodeling are changes in cardiac function, tissue structure, and size; the pathological changes include myocardial hypertrophy, apoptosis, re-expression of embryo genes and proteins, abnormal increase of non-cellular components (collagen fibers) and proportional changes (Burchfield et al., 2013; Xie et al., 2013). Ventricular remodeling is considered the basis for the development of heart failure, which determines morbidity and mortality in patients with heart failure. Therefore, delaying ventricular remodeling is the main direction for the treatment of heart failure. This study further demonstrated that perindopril had similar effects as Gal-3 inhibition, and could alleviate myocardial fibrosis by improving ventricular remodeling by downregulating Gal-3. These findings provide novel insights into the treatment of heart failure.

In fact, Gal-3 looks to play an active role from cardiac inflammation toward cardiac fibrosis, as a protein predominantly expressed by activated macrophages in patients with heart failure (Frunza et al., 2016). Consequently, I do not think that Gal-3 is implied in the myocardial infarction extension or in the alleviation of myocardial infarction. Moreover, other mechanisms are implied in myocardial extension that are differently expressed in different cases of myocardial infarction. As example, in the STEMI the number of coronary vessels and a pro-inflammatory/oxidative status might be involved in myocardial scar extension and future adverse cardiac events (Marfella et al., 2018b). Intriguingly, in the STEMI events the altered inflammatory/oxidative status (Marfella et al., 2018a), altered regenerative myocardial properties (Marfella et al., 2013b), and in addition pro-apototic and inflammatory atherosclerotic plaques characteristics (Balestrieri et al., 2015), such as a pro-thrombotic status (Sardu et al., 2018a) might represent multiple and different mechanisms involved in the concept that you were representing before as myocardial infarction extension, in the sense of anatomic entity of scar extension, myocardial damage, and cause of future worse prognosis. However, all these effects, from inflammation, toward myocardial fibrosis (and apoptosis), plus anti-regenerative muscle properties, have not presented in the current study. In my opinion all these points have to be clearly discussed, and presented as relevant study limitations.

The current study had limitations. First, MCP is a commonly used Gal-3 inhibitor in animal models, and has been shown to weaken inflammatory responses and fibrosis in several reports. However, Gal-3 inhibitors that could be used in clinical practice are rare, and need further exploration. In addition, the molecular mechanisms by which MCP and perindopril alleviate interstitial collagen deposition in the myocardium and inhibit myocardial fibrosis were not assessed comprehensively.

## Conclusion

The current study demonstrated that perindopril and MCP comparably improve cardiac function in rabbits with ischemic heart failure. These effects were reflected by reduced Gal-3, collagen type I, and collagen type III levels, resulting in decreased myocardial fibrosis, which could delay ventricular remodeling and prevent heart failure.

## Ethics Statement

This study was carried out in accordance with the recommendations of Ethics Committee on Scientific Research of Hebei General Hospital. The protocol was approved by the Ethics Committee on Scientific Research of Hebei General Hospital, Shijiazhuang, China.

## Author Contributions

ShuL conceived the study. ShaL and ShuL designed and supervised the study, acquired funding, provided materials, and critically reviewed the manuscript. ShaL, XH, YZ, and WD collected and processed the data. ShaL analyzed and/or interpreted the data, performed the literature search, and wrote the manuscript.

## Conflict of Interest Statement

The authors declare that the research was conducted in the absence of any commercial or financial relationships that could be construed as a potential conflict of interest.
